# Randomised controlled trial of cervical radiofrequency lesions as a treatment for cervicogenic headache [ISRCTN07444684]

**DOI:** 10.1186/1471-2253-6-1

**Published:** 2006-02-16

**Authors:** Sara RS Haspeslagh, Hans A Van Suijlekom, Inge E Lamé, Alfons GH Kessels, Maarten van Kleef, Wim EJ Weber

**Affiliations:** 1 Depts. Anesthesiology, Clinical Epidemiology, and Neurology, University Hospital Maastricht, The Netherlands

## Abstract

**Background:**

Cervicogenic headache (CEH) is a unilateral headache localised in the neck or occipital region, projecting to the frontal and temporal regions. Since the pathogenesis of this syndrome appears to have an anatomical basis in the cervical region, several surgical procedures aimed at reducing the nociceptive input on the cervical level, have been tested. We developed a sequence of various cervical radiofrequency neurotomies (facet joint denervations eventually followed by upper dorsal root ganglion neurotomies) that proved successful in a prospective pilot trial with 15 CEH patients. To further evaluate this sequential treatment program we conducted a randomised controlled trial

**Methods:**

30 patients with cervicogenic headache according to the Sjaastad diagnostic criteria, were randomised. 15 patients received a sequence of radiofrequency treatments (cervical facet joint denervation, followed by cervical dorsal root ganglion lesions when necessary), and the other 15 patients underwent local injections with steroid and anaesthetic at the greater occipital nerve, followed by transcutaneous electrical nerve stimulation (TENS) when necessary. Visual analogue scores for pain, global perceived effects scores, quality of life scores were assessed at 8, 16, 24 and 48 weeks. Patients also kept a headache diary.

**Results:**

There were no statistically significant differences between the two treatment groups at any time point in the trial.

**Conclusion:**

We did not find evidence that radiofrequency treatment of cervical facet joints and upper dorsal root ganglions is a better treatment than the infiltration of the greater occipital nerve, followed by TENS for patients fulfilling the clinical criteria of cervicogenic headache.

## Background

Cervicogenic headache (CEH) is a unilateral headache localised in the neck or occipital region, projecting to the frontal and temporal regions. CEH has been described as early as 1926 [1]. Sjaastad et al. were the first to give it its current name [2,3], and these authors formulated diagnostic criteria [4-6]. Although CEH as a separate diagnostic entity is still controversial [7], we found that one can reliably delineate CEH from primary headaches as migraine and tension-type headache [8]. The prevalence of CEH in the general population has been reported to be 0.4 to 2,5 % [6,9,10].

Since the pathogenesis of this syndrome appears to have an anatomical basis in the cervical region [11,12], several surgical procedures aimed at reducing the nociceptive input on the cervical level, have been tested. These include operative decompression of occipital nerves and cervical nerve roots [13-15], occipital neurectomy [16,17], cervical epidural injections [18], cervical manipulations [19-24], blockades of peripheral nerves with local anaesthetics [25-28], radiofrequency denervations of the periost of the occipital bone [29], botulinum toxin injections [30], and complementary therapies [31]. In a recent review of all treatments for CEH, Martelletti and van Suijlekom [32] concluded that consensus on a standard treatment for CEH does not exist, because of the great variability in patient selection and clinical effects. Since radiofrequency cervical facet joint denervations as an isolated treatment for CEH are often ineffective, we developed a sequence of various cervical radiofrequency neurotomies (facet joint denervation followed by dorsal root ganglion neurotomies) that proved successful in a pilot trial with 15 patients [33]. To further evaluate this sequential treatment program we conducted a randomised controlled trial.

## Methods

### Patient selection

The study was conducted in the departments of Pain Management and Research Centre and of Neurology of the University Hospital of Maastricht, The Netherlands. The University Hospital Maastricht Institutional Review Board (Research Ethics Committee) approved the study. All patients gave a written informed consent before entering the trial. The study group was recruited from patients with cervicogenic headache (CEH) according to the diagnostic criteria of Sjaastad [4]. We discussed the trial design extensively with colleagues of the department of Neurology, Trondheim University Hospital, Norway, and decided that the other following inclusion criteria had to be fulfilled: (1) age between 20 and 65 years; chronic cervicogenic headache[4] of more than 2 years' duration; (2) an initial visual analogue scale (VAS) score of more than 50 mm during a pain period; (3) a significant pain during at least two days per week. Excluded from the study were patients who had had previous surgical procedures of the cervical spine; who had coagulation disturbances; who were pregnant; who had multilevel severe degenerative changes at their cervical X-ray; who were diagnosed with post-whiplash syndrome. Patients matching the criteria for CEH were randomised into two groups: Group I was the RF-lesion group and Group II was the local injection group.

### Methods in Group I

Patients allocated in Group I first (*step 1*) had an RF-lesion of the zygoapophyseal joints at the levels C_3_–C_6 _at the affected side [33]. If -after 8 weeks- the facet denervation did not relieve the headache sufficiently (see below), the next step (*step 2*) was taken: diagnostic cervical segmental nerve blocks at the levels that were most likely to conduct an excess of afferent stimuli, were performed [34]. These levels were identified on physical examination, revealing tenderness at certain areas which are specific for a segmental level. At least two adjacent levels were tested at weekly intervals. When there was a reduction of at least 50 % of the VAS, an RF lesion adjacent to the relevant dorsal root ganglion (DRG) was performed [33,34]When there was no positive diagnostic block or when -after the RF lesion of the DRG and 8 weeks following the first step- there was no sufficient relief of the headache (see below), *step 3 *was undertaken: Transcutaneous Electrical Nerve Stimulation (TENS).

#### Technique of radiofrequency percutaneous facet denervation

The postero-lateral approach was chosen to perform an RF-lesion of the medial branches of the posterior primary rami of the facet joints C_3_–C_4_, C_4_–C_5 _and C_5_–C_6_. With the patient supine on the operating table, a C-arm image intensifier was positioned in a moderately oblique (+/- 30 %) position, until the projection of the pedicles were seen a little anterior to 50 % of the vertebral body. In the frontal plane, a small angle of the C-arm with the transversal plane was obtained to give a clear visibility of the intervertebral discs and the neuro-foramina. In this projection, the medial branch runs over the base of the superior articular process that is clearly visible. Entry points were marked on the skin, posterior and caudal to the target points as seen on the monitor after disinfection of the skin. A 22 SWG SMK C5 (Radionics, Inc., Burlington, MA) cannula with a 4-mm active tip was introduced at each entry point. First the needles were inserted at a maximal depth of 2 cm in a horizontal plane so that the needle points were in line with their target points. Subsequently, each needle was carefully advanced anteriorly and cranially until bone contact was made with the facetal column at the target point. The position of the needle points was than checked in the antero-posterior plane: the needle tips were supposed to project adjacent to the concavity of the articular pillars of the cervical spine at the corresponding levels. When an optimal anatomical position of the needle was reached, this was confirmed by electrical stimulation. The 50 Hz threshold should elicit a response (tingling sensation) in the neck at < 0,5 Volt and the 2 Hz stimulation should confirm no muscle movements in the ipsilateral shoulder and/or arm. Subsequently, 1 ml of local anaesthetic solution (Lidocaine 2%) was given at each level and a 60-sec 67°C lesion was made.

#### Technique of diagnostic block and radiofrequency lesioning of the cervical segmental nerves

The patient was lying supine and the C-arm was positioned obliquely until the contralateral pedicles were projecting posterior to the anterior line of the vertebral bodies. In the frontal plane the C-arm was adjusted until the intervertebral discs were clearly visible. The target point was posteriorly in the neuroforamen between the caudal and the middle third part. The entry points were marked on the skin and were the same as the entry points. One ml of local anaesthetics (Lidocaine 2%) was injected in the skin at each entry point and then an RCN-needle (Radionics. Burlington, MA) was introduced in a tunnel vision technique. Every step was checked in the lateral position where the needle tip was supposed to project 1 to 2 mm laterally to the lateral border of the facet column. After confirming the needle position close to the segmental nerve with water soluble contrast dye (Iohexol), 0.5 ml of local anaesthetics (Lidocaine 2%) was injected. After one or several positive diagnostic blocks, RF lesioning of the DRG of the involved cervical segmental nerves was performed. The technique was similar to the technique of the diagnostic blocks as described above. The differences were: another needle was used (a 22 SWG SMK C5 cannula with a 4-mm active tip, Radionics), the needle tip was advanced until it was projecting in the middle of the facet column (to reach the dorsal root ganglion of the segmental nerve) and before the RF lesioning, the 50 Hz and 2 Hz thresholds were looked for. The 50 Hz threshold should be between > 0, 4 and < 0,65 Volt. The 2 Hz threshold should not occur below a voltage of 1,5 times the 50 Hz threshold. If this was confirmed, 1 ml of Lidocaine 2% was injected and a RF-lesion was made.

### Methods in Group II

The *first step *in Group II was injection with local anaesthetics of the major occipital nerve on the affected side. If this therapy didn't relieve the headache sufficiently after 8 weeks, the treatment was repeated in *step 2*. If there was still no sufficient pain relief after 16 weeks, *step 3 *was undertaken: TENS-therapy.

#### Technique of blocking the greater occipital nerve (GON)

The needle was placed 2 cm. lateral and 2 cm. inferior to the external occipital protuberance [35]. The needle was first forwarded onto the periosteum of the occipital bone and was then withdrawn approximately 0,5 cm. before injection of local anaesthetic solution. After negative aspiration, 2 ml. of Bupivacaine 0,5 % was injected.

### Evaluation

An investigator blinded to the subjects' condition evaluated the patients. This investigator (IEL) did not take part in the actual treatment process and was thus not aware of the treatment that the patient had received. Patients were aware of this and were asked not to mention their received therapy. As we did not check the blinding efficacy, we cannot rule out that some unblinding of the investigator actually took place. We were under the impression that IEL remained blinded for treatment allocation throughout the trial. Rating of pain was evaluated by averaging three daily measurements of the VAS (Visual Analogue Score), ranging from 0 to 100 mm, during one week. We obtained these ratings from the diary that the patients kept trouhgout the trial. This mean VAS of one week was used in the evaluation of the effect of the treatment (VAS difference between the different weeks of the treatment). Global perceived effect (GPE) was scored by the patient on a 7-point scale (ranging from much worse: -3, to 0: no change, to total pain relief: +3). A sufficient relief of the pain indicated a mean VAS reduction of minimal 20 mm and/or a good result on the 7-point scale (i.e. complete relief: +3 or much better: +2). The number of headache days, the medicine use and the headache intensity during a week were also recorded. Follow-up was done by an independent neurologist (WW) who performed a physical/neurological examination at every step of the study. The patient was also asked to fill in questionnaires: the RAND-36 [36,37], the MPI-DLV (Multidimensional Pain Inventory in the Dutch Language) [38,39] and the Dutch version of the SCL-90 [40,41]. The patients were assessed 4 weeks before treatment and 8 weeks after treatment. Treatment was scored as a success, if there was a reduction of the mean VAS of at least 2 points and/or a global perceived effect of +2 or +3. Further assessment was conducted at 4, 6, 8, 10 and 12 months after treatment. If failure occurred, the study provided a "next step" treatment after 2, 4 and 6 months after the initial treatment to avoid the unethical situation of not treating a patient with pain.

### Statistical analysis

The primary endpoint was the percentage of success at 8 weeks (**T1**). To compare the success rates between the lesion and the local injection groups, the Chi-square and if appropriate the Fisher exact tests were performed. P values = 0.05 were considered statistically significant. Secondary, the differences between the lesion and the local injection groups in VAS scores, percentage VAS improving, mean days of headache, headache intensity and quality of life domain scores at 8, 16, 24 and 48 weeks were compared, using a Student's t test.

## Results

### Demographic and clinical characteristics

From September 1997 until June 2002, 112 consecutive patients with headache were screened of whom 65 had Cervicogenic Headache (CEH), according to the criteria [42]. 35 patients did not enter the study for the following reasons: 26 did not fulfil the selection criteria and 9 refused to participate in the study. Thirty patients entered the study and were randomised.

The patient baseline characteristics and psychometric properties of the Radiofrequency group (Group I) and of the Local injection group (Group II) are shown in Table 1. There were no significant group differences in these variables. The "mean VAS a week" was the mean VAS of the last 7 days (measured three times a day) before entering the trial. The "days headache a week", the "medicine use a week" and the "headache intensity a week" were the averages over the last 4 weeks before entering the trial. The Rand-36 represents the patients' health concerning the Physical and Social Function (PF and SF), the Role Physical and Role Emotional Limitations (RP and RE), the Mental Health (MH), the Vitality (VI), the Bodily Pain (BP) and the General Health (GH). These variables were not significantly different between both groups.

**Table 1:** Patient baseline characteristics and psychometric properties

	***RF (Group I)***	***Control (Group II)***
Number of patients (n)	15	15
Mean age (SD) [min/max] (yr)	47,5 (11,0) [22/62]	49,1 (12,8) [28/64]
Male/female (n)	4/11	4/11
Duration of pain (yr)	9,7	6,6
SCL-90 psychoneurotism (SD)	146,3 (29,8)	135,4 (25,0)
Mean VAS/4 weeks (SD)	68,1 (12,7)	76,5 (16,6)
Days headache/4 weeks (SD)	25,9 (5,0)	19,0 (9,3)
Medicine use/week (SD)	6,7 (5,0)	5,8 (8,3)
Headache intensity/week (SD)	2,1 (0,4)	1,9 (0,4)
		
Rand-36 (SD):		
*PF*	70,0 (21,4)	57,0 (24,6)
*SF*	71,7 (18,0)	59,2 (23,4)
*RP*	31,7 (34,7)	36,7 (35,2)
*RE*	64,4 (38,8)	66,7 (35,6)
*MH*	65,3 (16,2)	69,6 (16,8)
*VI*	53,7 (24,3)	45,3 (15,2)
*BP*	41,8 (19,4)	38,1 (18,5)
*GH*	58,7 (21,0)	54,7 (18,5)
		
MPI (SD):		
*Pain severity*	45,6 (9,6)	36,8 (13,6)
*Interference*	42,0 (14,1)	44,6 (15,9)
*Life control*	55,0 (8,4)	58,0 (9,2)
*Affective distress*	48,0 (9,6)	41,9 (9,8)
*Support*	52,5 (9,8)	51,8 (8,6)
*Punishing responses*	48,1 (9,5)	45,5 (7,1)
*Solicitous responses*	53,3 (10,8)	51,3 (10,4)
*Distracting responses*	50,4 (13,2)	46,5 (9,4)
*Household chores*	48,4 (9,9)	48,5 (11,1)
*Outdoor work*	53,5 (9,4)	56,5 (15,9)
*Social activities*	54,4 (12,0)	52,7 (12,0)
*General activity*	52,8 (12,0)	53,9 (12,0)

### randomisation and follow-up of the study (Figure 1) (Table 2)

**Figure 1:**
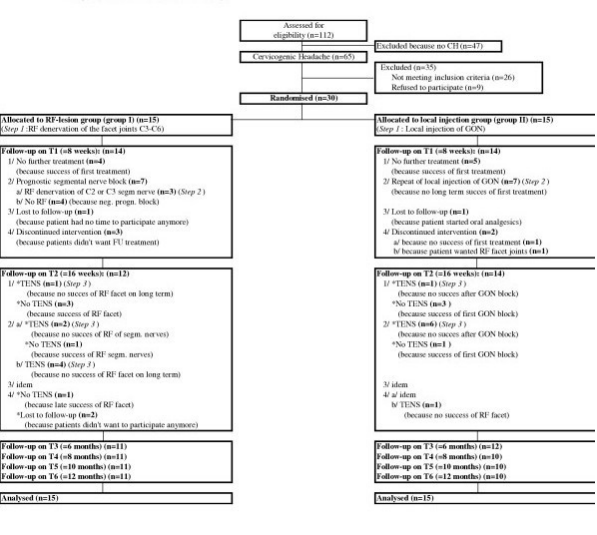
Flowchart of the study.

**Table 2:** Randomisation and follow-up of the study patients

	**T0 ***Step I*	**T1 (= 8 weeks) ***Step II*			**T2 (= 16 weeks) ***Step III*		
**Patient number**	**Randomization**	**GPE**	**VAS success**	**Follow-up treatment**	**GPE**	**VAS success**	**TENS**

1	GON	0	_	GON	-1	_	Yes, -
2	PFD C3–C6	0	_	Patient does not want follow-up treatment	Missing	+	No
3	GON	+2	_	No	0, again GON	_	No
4	PFD C3–C6	+1	+	Prog C2, prog C3 Policy: RF C2	Missing	+	No
5	PFD C3–C6	-1	+	Patient does not want follow-up treatment	OS	OS	OS
6	GON	-1	+	GON	1	+	No
7	GON	0	+	Heart catheterization, no follow-up treatment	2	_	No
8	PFD C3–C6	+2	_	No	1	+	No
9	PFD C3–C6	0	_	Progn. C2, C3, C5: all no effect	0	+	Yes, -
10	GON	+1	+	GON	2	_	Yes, +
11	PFD C3–C6	+1	+	Prog C2, prog C3 Policy: RF C2	1	+	Yes, +
12	GON	+2	+	No	2	+	No
13	PFD C3–C6	-2	_	Yes, but patient had no time to come anymore	OS	OS	OS
14	PFD C3–C6	+2	_	No	2	Missing	No
15	GON	+3	Missing	No	2	_	No
16	GON	+1	+	GON	0	+	Yes, +
17	GON	-2	_	GON	1	Missing	Yes, +
18	GON	0	+	PFD C3-C6	0	+	Yes, -
19	PFD C3–C6	-2	+	OS, not very content with the treatment	OS	OS	OS
20	PFD C3–C6	+2	+	No	-2	_	Yes, +
21	GON	+2	+	No	2	+	No
22	PFD C3–C6	0	+	Progn. C2, progn. C3: all no effect	1	+	Yes, -
23	PFD C3–C6	+2	+	No	2	+	No
24	GON	+1	+	GON	0	_	Yes, OS
25	PFD C3–C6	0	+	Progn. C2, C3, C4: all no effect	0	+	Yes, -
26	GON	OS	Missing	OS	OS	Missing	OS
27	PFD C3–C6	0	+	Progn. C2, C3. Policy: RF C3	+1	+	Yes, -
28	GON	+1	_	GON	-2	_	Yes, +
29	GON	-2	_	OS, does not want any treatment	OS	OS	OS
30	PFD C3–C6	0	+	Progn. C2, C3: all no effect	-2	_	Yes, +

GON = Greater Occipital Nerve

PFD = Percutaneous Facet Denervation

GPE = Global Perceived Effect (-3 = much worse, -2 = worse, -1 = little worse, 0 = no effect, +1 = improved, +2 = much improved, +3 = no complaints anymore)

VAS = Visual Analogue Score (+ = success, - = no success)

OS = Off Study

TENS = Transcutaneous Electrical Nerve Stimulation (+ = success, - = no success)

Eight weeks (**T1**) after the initial treatment, the patient received a follow-up treatment when the global perceived effect was 0 or less than zero (no effect:0, little worse:-1, worse: -2 or much worse:-3). The VAS success wasn't a crucial parameter in making the decision to continue the next radiofrequency treatment (Group I) or to repeat the local injection of the greater occipital nerve (Group II).

#### Group I

There was 1 patient (number 13) in group I who dropped out of the study on **T1 **because he refused further participation in the study, despite of the fact that the GPE was -2 and thus a follow-up treatment was suggested. In the evaluation of the effect at the primary endpoint, he was categorized as a negative responder (see negative VAS and/or GPE of -2 on **T1**). There were 4 patients (patient numbers 8, 14, 20 and 23) who did not receive a follow-up treatment because of a positive GPE (+2) on **T1**. Two of them (patient number 14 and 23) continued to have a positive GPE (+2) after 16 weeks (**T2**). There were another 3 patients (patient numbers 2, 5 and 19) in the RF group who did not want a follow-up treatment after 8 weeks (on **T1**). Patient number 2 did not want a follow-up treatment in spite of a negative global perceived effect (GPE = 0) and a negative VAS-success on **T1**. The patient only filled in the pain diary which revealed a positive VAS-success after 16 weeks (**T2**) without any follow-up treatment. The global perceived effect was missing. He was categorized as a positive responder in the evaluation of the primary endpoint. Patient number 5 and patient number 19 dropped out of the study after 8 weeks because they were disappointed with the treatment. They both had a negative global perceived effect (-1 for patient 5 and -2 for patient 19) despite of the fact that both their VAS successes were positive. In the evaluation they were accepted to be positive responders (since they had a positive VAS on **T1**). There were 7 patients (patient numbers 4, 9, 11, 22, 25, 27 and 30) who received at least two diagnostic segmental blocks of cervical nerves (C_2_, C_3 _and seldom others). Of them, 4 patients (patient numbers 9, 22, 25 and 30) were not treated with a radiofrequency lesion of a cervical segmental nerve because of negative diagnostic blocks. Patient number 4 was treated with an RF of the DRG of C_2 _and patient numbers 11 and 27 were treated with RF of the DRG of C_3 _because of positive diagnostic blocks. None of them reported a positive GPE after 16 weeks (**T2**), but they all reported a positive VAS success. In the evaluation of the effect at the primary endpoint (**T1**), patient number 9 was classified as non-successful (GPE of 0 and negative VAS success on **T1**) and patients numbers 22, 25 and 30 were called to be successful (they all reported a positive VAS success on **T1**).

#### Group II

In the local injection group (Group II) there was one patient (patient number 26) who did not show up on **T1 **and on later evaluations. He dropped out because he started oral analgesics that reduced his pain significantly. He was categorized as "off study" (OS). Furthermore there were 7 patients (patient numbers 3, 7, 12, 15, 18, 21 and 29) in this group that did not receive a second local injection of the greater occipital nerve on **T1**. Four of them (patient numbers 3, 12, 15 and 21) had a GPE of +2 or +3 and therefore a follow-up treatment was not given in the study protocol. Of the other 4 patients, one patient (number 3) asked for a second local injection treatment 16 weeks after the initial treatment (on **T2**) because his global perceived effect was decreased from +2 (on **T1**) to 0. In the evaluation of the effect on the primary endpoint, he was considered a treatment success The other three patients (patient numbers 12, 15 and 21) who did not receive a second local injection of the greater occipital nerve, because they all had a positive GPE (+2) on **T1**, reported a long-lasting positive effect (GPE +2 after 16 weeks (**T2**)). Of the other 3 patients (patient numbers 7, 18 and 29) that did not receive a second local injection of the greater occipital nerve, despite of the fact that they reported a negative GPE (0 or -2), one patient (patient number 7) underwent a heart catheterization because of occlusive coronary vascular disease. Nevertheless, he continued to fill in the pain diary and the question lists and was thus included in the final evaluation of the technique and he was categorized as a positive responder (VAS success + on **T1 **and GPE = +2 on **T2**). Another patient (patient number 29) didn't want a follow-up invasive treatment anymore despite of the negative GPE (-2) and the negative VAS success after 8 weeks (**T1**). He dropped out because he was not very content with the first treatment. In the final evaluation of the technique, he was categorized as negative responder (since he had a negative VAS success and a GPE of -2 on **T1**). Patient number 18 did not believe in the local injection technique despite of the positive VAS success on **T1 **and demanded a percutaneous facet denervation of the C_3 _to C_6 _facet joints on **T1**. This patient was included in the evaluation of the local injection group (*Group II*) after 8 weeks, but was excluded in the further evaluations of the local injection technique because of his cross-over to the RF treatment. Seven patients of the local injection group (patient number 1, 6, 10, 16, 17, 24 and 28) received a second local injection of the greater occipital nerve on **T1 **because of a "negative" GPE (i.e. +1, 0, -1 or -2). Patient number 1, 17, 24 and 28 reported a negative outcome on **T2 **after the repeated local injection. They were assessed to try out TENS. The other 3 patients (patient number 6, 10 and 16) reported or a positive GPE (patient number 10) or a positive VAS-success (patient number 6 and 16) on **T2**. Patient number 6 did not try out TENS, patient numbers 10 and 16 tried out the TENS although they reported a positive GPE (+2 for patient number 10) or a positive VAS success (patient number 16).

### Difference in VAS, days of headache and intensity of headache of both groups compared to the initial values (Table 3)

**Table 3:** VAS, quantity of days of headache and intensity of headache at different timesin the study (T1, T2, T3 and T6) compared with T0

	***RF (Group I) ****Mean (SD)*	***Control (Group II) ****Mean (SD)*	***p***	***95% CI***
VAS difference				
*T1-T0*	30,5 (17,3)	32,4 (24,7)	0,81	-14,4 to 18,3
*T2-T0*	29,9 (13,8)	21,0 (35,5)	0,41	-31,2 to 13,5
*T3-T0*	28,9 (20,3)	24,6 (35,0)	0,69	-26,6 to 17,9
*T6-T0*	30,2 (12,4)	26,8 (37,7)	0,75	-25,8 to 19,1
Headache difference				
*T0 T1*	4,2 (5,1)	5,5 (8,7)	0,62	-4,3 to 7,1
*T0 T2*	4,1 (4,1)	3,9 (6,3)	0,94	-4,5 to 4,2
*T0 T3*	7,5 (7,1)	4,5 (6,1)	0,27	-8,3 to 2,4
*T0 T6*	5,6 (5,7)	6,8 (7,7)	0,65	-4,2 to 6,7
Intensity difference				
*T0 T1*	1,5 (4,0)	- 0,5 (8,7)	0,43	-3,1 to 7,1
*T0 T2*	2,3 (4,2)	-1,0 (9,6)	0,23	-2,2 to 8,9
*T0 T3*	3,1 (4,5)	- 0,6 (9,2)	0,18	-1,8 to 9,2
*T0 T6*	3,7 (8,7)	- 0,4 (9,4)	0,24	-2,9 to 10,9
Percentage VAS improving				
*T1*	43,9 (22,0)	42,4 (28,6)	0,87	-21,2 to 18,1
*T2*	45,4 (23,9)	24,1 (50,1)	0,17	-52,0 to 9,5
*T3*	41,7 (28,5)	28,0 (49,4)	0,38	-45,0 to 17,6
*T6*	44,4 (16,8)	30,7 (49,9)	0,34	-43,5 to 16,1

VAS difference = mean difference of VAS on T1, T2, T3 and T6 compared with T0 in mm mean VAS is the mean VAS of one week (three times a day) before T1, T2, T3 or T3

Headache difference = mean days of headache 4 weeks before T1, T2, T3 and T6 compared with mean days of headache before T0

Intensity difference of headache = mean days of heavy pain intensity 4 weeks before T1, T2, T3 and T6 compared with heavy pain intensity before T0.

Percentage VAS improving = mean VAS improved on T1, T2, T3 and T6 compared with T0 in %.

The VAS difference between the different moments (T1, T2, T3 and T6) compared to the initial VAS (T0) showed an improvement at every moment in the study in each group. Between both groups there was no significant difference in improvement.

The amount of headache days on the different moments (T1, T2, T3 and T6) compared to T0 decreased during the study and this was not significantly different in Group I compared to Group II.

The difference in headache intensity in which the intensity of the headaches on the different moments (T1, T2, T3 and T6) where compared to the headache intensity before T0, seemed to be better at each moment in Group II compared to Group I, although these differences where not significant!

In the RF-group the percentage of improved VAS compared to T0 was not significantly different between the different moments, namely between 41,7 and 45,4%. In Group II, the percentage of the improved VAS was the highest on T0/T1 (42,4 %) compared to the lowest percentage of improvement on T0/T2 (28,0 %). These outcomes where not significant different. Furthermore, there was no significant difference in VAS improvement between both groups.

### Positive global perceived effects (GPE of +2 or +3) and/or a successful VAS-difference (Table 4)

**Table 4:** Number of patients with a positive GPE and/or a successful VAS at different times in the study (T1, T2 and T6).

	T1 (= 8 weeks)			T2 (= 16 weeks)			T6 (= 1 year)		
	***Group I***	***Group II***	*Total*	***Group I***	***Group II***	*Total*	***Group I***	***Group II***	*Total*
n with success (%)	12 (80%)	10 (66,7%)	22 (73,3%)	10 (66,7%)	8 (53,3%)	18 (60%)	8 (53,3%)	7 (46,7%)	15 (50%)
n no success (%)	3 (20%)	4 (26,7%)	7 (23,3%)	2 (13,3%)	5 (33,3%)	7 (23,3%)	2 (13,3%)	3 (20%)	5 (16,7%)
n no data (%)		1 (6,7%)	1 (3,3%)	3 (20%)	2 (13,3%)	5 (16,7%)	5 (33,3%)	5 (33,3%)	10 (33,3%)

Group I = Radiofrequency treatment group

Group II = Local injection group

Eight weeks after the initial treatment (**T1**), 80% of the patients in the RF-group (Group I) and 66,7% of the patients in the local injection group (Group II) reported a successful treatment in terms of a positive global perceived effect and/or an VAS reduction of at least 50% compared to the initial VAS. This meant no statistically significant difference in success rate between both groups.

Sixteen weeks after the initial treatment (**T2**), the success rate in Group I was 66,7% compared to 55,3% in Group II. Again, this difference is not statistically significant. After one year (**T6**), there was no difference of the success rate in Group I (53,3%) compared to Group II (50%). A relatively high percentage of patients (33,3%) in both groups were not followed anymore because of several reasons. The most important reason was the disappointment in the treatment.

### Mean health scores in the RF-group (Group I) and the GON-group (Group II) at different times (T0, T1, T2 and T6) (Figure 2)

**Figure 2:**
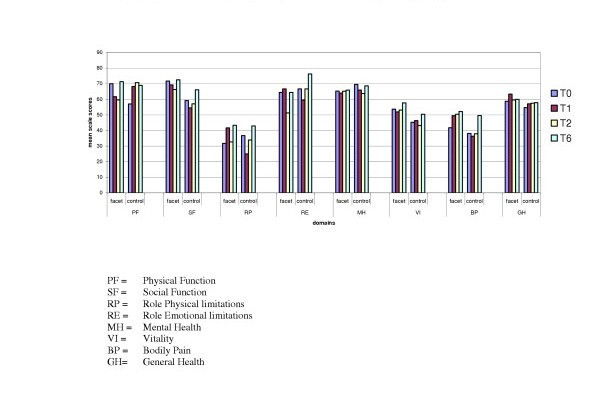
Comparison of mean health domains at different follow-up measurements for both groups (I = Radiofrequency Group and II = Local injection group).

This figure shows no significantly difference between the mean health scores of both groups at different moments.

## Discussion

The primary goal of this study was to compare the efficacy of a radiofrequency treatment with a treatment by local injection of the greater occipital nerve in patients with cervicogenic headache (CEH). Although CEH is a controversial diagnosis[7], it is a widely used diagnosis for which radiofrequency treatment is given in routine clinical care. As the scientific basis for this therapy is also controversial, we sought to restrict ourselves to a clearly defined headache syndrome. We demonstrated earlier that CEH can be relatively reliably delineated from the other primary headaches[8]. We feel that the rigorous selection of patients such as we did for this trial (see below) led to a rather homogenous group of CEH patients. These patients, with a strictly unilateral headache without side-shift and pain originating in the neck, were shown by Antonaci et al[43]to be most reliably diagnosable as CEH. Most of these would proably also fulfill the International Headache Classification criteria for CEH[44]. Much effort was spent to select patients with CEH according to recent criteria[42]; we screened 112 patients, of which only 30 entered our study.

We thus randomised 30 patients with cervicogenic headache into two groups receiving either treatment. In the different steps of the study, the patients could get a follow-up treatment if the first treatment was not successful or only had a short period of success. We undertook this study, encouraged by the success of our earlier open trial with good results after long-term follow-up [33] Because of the setup of the trial (we compared two possible sequences of treatments, rather than two isolated ones), conclusions on isolated treatment effects cannot be made. But we may conclude that a sequence of possible radiofrequency treatments (beginning with a cervical facet joint denervation) is not better than a more conservative sequence of therapies including local injections and TENS. This is reflected in the primary outcome measure (pain and global perceived effect at 8 weeks) and also in the secondary outcome parameters, at any given time point in the study. The positive effects we observed in our earlier open study [33], probably reflects the less rigorous methodology of that trial. In fact we have observed a similar phenomenon (less clinical effect with better trial methodology) when we studied the effects of intradiscal radiofrequency therapy for low back pain [45,46]. Because of the lack of difference between the two treatment groups, we feel that the blinding of the evaluating investigator in the present study was relatively good, as one would expect a possible bias to emerge preferably in the more invasively treated group.

To this date only one other randomised controlled trial was undertaken to study the efficacy of a radiofrequency denervation of cervical facet joints in patients with cervicogenic headache [47]. They randomised patients in two groups of each 6 patients to receive an RF neurotomy of facet joints C2 C6 ipsilateral to the pain, or a sham treatment. They found a minor improvement in patients treated with an RF denervation at 3 months, but later on there were no marked differences between groups.

## Conclusion

We did not find evidence that RF treatment of cervical facet joints and dorsal root ganglion is an effective treatment for patients fulfilling the clinical criteria of cervicogenic headache. We do share the concern of Stovner et al that many such patients are treated with these neurotomies despite lack of robust evidence for positive effects [47].

## Competing interests

The author(s) declare that they have no competing interests.

## Authors' contributions

Hans A. Van Suijlekom and Wim E.J. Weber initiated the trial; they, with Alfons G.H. Kessels and Maarten van Kleef wrote the study protocol. HAVS, WEJW, MVK and Inge E. Lamé conducted the study. Data collection and interpretation was done by HAVS, WEJW and Sara R.S. Haspeslagh. Statistical analysis wad done by IEL and AHFK. SRSH wrote the first draft of the manuscript that was finished in its final form by WEJW.

## Pre-publication history

The pre-publication history for this paper can be accessed here:


